# Influence Factors on Birth Spacing and Childbearing Rates using Survival Recurrent Events Model and Parity Progression Ratios

**Published:** 2017-07-07

**Authors:** Roya Najafi-Vosough, Ali Reza Soltanian, Naser Fayyazi

**Affiliations:** ^1^ Department of Biostatistics, School of Public Health, Hamadan University of Medical Sciences, Hamadan, Iran; ^2^ Modeling of Noncommunicable Diseases Research Center, Hamadan University of Medical Sciences, Hamadan, Iran; ^3^ Department of Management & Health Economic, School of Public Health and Modeling of Noncommunicable Diseases Research Center, Hamadan University of Medical Sciences, Hamadan, Iran

**Keywords:** Survival analysis, Reproductive, Birth interval

## Abstract

**Background:** Reproduction rates are declining in Hamadan, western Iran. This study identified the influence factors associated with reproduction rate and birth spacing in Hamadan as an area of low population growth in Iran.

**Study design:** A cross sectional study.

**Methods:** The study considered reproductive status of 812 women referred to health care centers of Hamadan, western Iran in 2015. Data were obtained through frequency and percentage. PWP-GT model was used to determine the influence factors on women's reproductive by R software (version 3.3.2). The parity progression ratios were determined based on Yadava and Kumar.

**Results:** The median time for the second, the third and the fourth birth was 4.53, 4.65 and 5.27 yr, respectively. PWP-GT model showed that women age at marriage (*P*=0.001), women's (*P*=0.005) and their husband's (*P*=0.039) employment had significant effect on time birth of first child. The women's education (*P*=0.001) was the only variable that had a significant effect on the birth time from the first to the second child as well as from the third to the fourth child. Education of women (*P*=0.001) and their husbands (*P*=0.034) had significant effect on the time interval from the second to the third child. The birth probability from marriage to the first birth (0 to 1st child) was high (0.91), while the probability had been fallen from the third birth and more (0.31).

**Conclusions:** Birth spacing in Hamadan is more than WHO recommended. In addition, reducing of the population growth and childbearing has started in the past few decades in Hamadan the same as Iran. The chance of fertility has dramatically declined from the third child and then.

## Introduction

The structure of populations will change over time by fertility, mortality, and migration. Then, determination of birth rate, birth spacing, and its influence factors are important. WHO recommends that all countries to apply demographic information such as registration of current events and in economic planning^1^. Knowing the average of family size and parity progression ratios (PPRs) are the most important information on population policies^2^, so that rise and fall of the PPRs indicate transmission of the population. Therefore, study and continuous tracking of changes in fertility and its components is important in each geographic area.

Iran's strategy has focused on the prevention of pregnancy in past two decades^3^; but now, has been faced with reduction population growth. The fertility rate of Iranians' women in 2016 (i.e., 1.72) was approximately three times smaller than 1975 (i.e., 6.24)^4^. Hence, research for the fertility status of women in the past two decades and even before, could be interesting. In addition, such studies can show the impact of demographic policy of the government in reducing population growth.

In Iran, parity progression ratio from first birth to the second birth and more was relatively high, and several demographic factors have influenced on it^5^. Hamadan Province is ninth for total fertility rate in Iran^6^. Furthermore, according to the Statistical Center of Iran report, Hamadan Province was among the areas with the lowest population growth in the West of Iran^7^. Population growth in Hamadan Province has fluctuated over the years 2005 to 2010, and has not a clear increase or decrease trend^8^. However, detailed results among previous censuses show that growth of birth registered in Hamadan Province has a decreasing trend^8^. Continuing the downward trend in the long term may reduce population growth in this province. Total fertility rate in the province was 1.6 in 2009, smaller than a whole country^8^. The most influence factors in reducing fertility rate are may be birth spacing, high literacy^9, 10^, higher marriage age^9, 11^, the employment situation couples^5, 12^ and other demographic characteristics.

Therefore, the study on the dynamics of demography in Hamadan is necessary. The purpose of this study was to model the factors related to the birth spacing in Hamadan. In this study, the dynamic demographic indicators, especially parity progression ratios (PPRs) which reflect the status of increasing/decreasing household size, were determined.

## Methods

### Data collection

This cross-sectional study was approved by the Ethics Committee of Hamadan University of Medical Sciences, Hamadan, Iran.

The study considered reproductive status of 812 women in Hamadan, western Iran in 2015. Women participating in the study must have had at least one legal marriage, 45 yr or older, and had been living in Hamadan. Participants were selected by the two-stage sampling method. In the first stage, 15 centers (50%) from 32 Hamadan’s health care centers were randomly selected. In the second stage, six heads of the cluster were randomly selected from the list of households in these centers.

### Statistical Analysis

Recurrent childbirth modeling: As for the repetitive nature of childbirth, women may experience its several times in their lifetime. The time interval between deliveries can be analyzed using models for recurrent events. In these models, the given event (i.e., childbirth) occurs more than once for each individual does. To investigate recurrent events, selection of the appropriate model depends on the research objective, researcher, and the nature of the data.

In the present study, the Prentice-Williams-Peterson gap time (PWP-GT) conditional model was used to evaluate different influence factors on the time interval between deliveries. The follow-up time in each event reset to zero and the time interval between the two events was considered as birth spacing.

The hazard function model for the ith individual is:

[1] $$h(t|b_{s},X_{i}(t))=h_{0s}(t-t_{s-1})\exp[{b^{\prime }}_{s}X_{i}(t)]$$

where, *s* is the number of strata for each person at time t, *X*_i_*(t)* denotes the predictor variable for individual *i* at time t, and *t*_s-1_ denotes the former occurrence time of the event.

The occurrence time of the event for the individual *l* in the *s* stratum is shown by *t*_sl_.The time interval equals:

usl=t−slt(s−1)l

And *t*_(s-1)l_ is the occurrence time for the individual *l* at *s-1* stratum.

The partial likelihood function for the PWP-GT model based on the time interval between the events in each stratum *(u_s(l))_<u_s(2)_<...<u_ s(d_s_)_* is,

[2] $$L(b)=\prod_{s\geq 1}\prod_{i=1}^{d_{s}}\frac{\exp[{b^{\prime }}_{s}X_{si}(t_{si})]}{\sum_{l\in \tilde{R}(u_{s(i)},s)}\exp[{b^{\prime }}_{s}X_{sl}(t_{si})]}$$

where *d*_s_ is the number of events in s stratum, *X*_si_*(t*_si_*)* is the predictor variable for the *i* individual in the *s* stratum in the time* t*_si_ , and $\tilde{R}(u_{s(i)},s)$indicates the at-risk individuals in the first stratum *s* before the time interval *u*^13^.

Parity Progression Ratios (PPRs): The PPR is the proportion of women with a certain number (i.e., *i*^th^ children) of children who go onto have another child (i.e., *(i+1)*^th^ children). The parity progression ratios is were determined based on Yadava and Kumar (4) as follows,

[3] $$a_{i}=\frac{c_{1}-p_{i}{}^{\left(o,c_{1}\right)}.c}{p_{i}{}^{\left(o,c_{1}\right)}[E_{c}(T_{i}*)-c]}$$

where, p_i_^ 0,C_1_^ is the proportion of females at i_th_ parity with open birth interval in the range zero to C_1_. Also C and C are the measures under open and closed birth intervals. Generally C and C_1_ were considered larg (e.g. more than 60) and 12 months. In addition $E_{c}(T*)=\int_{0}^{c}[1-F_{i}(t)]dt$, where, F_i_(t) denotes the distribution function of closed birth interval of females at i_th_ parity. In this study we considered C_1_=12 and C_1_=60, 72, 84, 96, 108, and 120 month. Therefore, the expression [3] is rewrite as follows,

[4] $$a_{i}=\frac{12-p_{i}{}^{\left(o,12\right)}.c}{p_{i}{}^{\left(o,12\right)}[E_{c}(T_{i}*)-c]}$$

Description of the collected information was performed through frequency and percentage, while data analysis was conducted using the PWP-GT model with the R software (version 3.3.2). The statistical significance level was less than 0.05.

## Results

The parity progression ratios and influence factors on recurrent birth child were assessed for 812 women, aged 45 yr or older. The minimum and maximum number of children for the women was zero to seven, respectively. However, the study did not go beyond fifth labor due to the small sample size. Of 812 women, 770 (94.83%) had only one child, 671 (82.63%) had two children, 254 (31.28%) had three children, and 73 (8.99%) had four children. In addition, the first infertility rate was 5.17%. The mean±sd age of the women and their husbands at the first delivery was 22.36±4.58 and 27.66±5.31 yr, respectively. The sex ratio at first birth was 104. Median of birth interval at the second, the third, and the fourth children was 4.53, 4.65 and 5.27 yr, respectively. Table 1 shows the frequency distribution of the women’s deliveries by demographic characteristics separately.

**Table 1:** The frequency distribution of the women’s deliveries by demographic characteristics

**Variables**	**First delivery** **(n= 770)**		**Second delivery** **(n= 671)**		**Third delivery** **(n= 254)**		**Fourth delivery** **(n= 73)**	
	**n**	**%**	**n**	**%**	**n**	**%**	**n**	**%**
Women's educational level								
Illiterate or primary	68	8.88	68	10.13	60	23.62	31	42.47
Secondary	284	36.88	270	40.24	139	54.72	33	45.20
High school	269	34.93	222	33.08	45	17.72	5	6.85
Academic	149	19.35	111	16.54	10	3.94	4	5.48
Women's employment status								
Employed	130	16.88	107	15.95	43	16.93	13	17.81
Unemployed	640	83.12	564	84.05	211	83.07	60	82.19
Husband's educational level								
Illiterate or primary	65	8.41	64	9.54	54	21.26	27	36.99
Secondary	367	47.66	470	70.04	174	68.50	25	34.25
High school	199	25.84	120	17.88	21	8.27	21	28.76
Academic	181	23.51	17	2.53	5	1.97	0	0.00
Husband's employment status								
Employed	758	98.44	660	98.36	246	96.85	66	90.41
Unemployed	12	1.56	12	1.79	8	3.14	7	9.59
Stillbirth								
Yes	30	3.89	24	3.58	16	6.29	5	6.85
No	742	96.36	647	96.42	238	93.71	68	93.15
First pregnancy (yr)								
≤18	163	21.17	123	18.33	109	42.91	44	60.27
>18	607	78.83	548	81.67	145	57.08	29	39.73

The results of the PWP-GT model fitting revealed that maternal age, mother and father employment status were significantly effective on the birth interval from marriage to the first child, so that increased maternal age at marriage was associated with reduced child birth ratio (HR=0.96, *P*<0.001, Table 2). In addition, the childbirth ratio of employed mothers was less than the unemployed ones (HR=0.685, *P*=0.005, Table 2). Hazard rate of having the first child in unemployed fathers was approximately 0.88 times lower than the employed ones (*P*=0.039, Table 2).

**Table 2:** The impact of demographic characteristics on childbirth by PWP-GT model

**Variables**	**First delivery**			**Second delivery**			**Third delivery**			**Fourth delivery**		
	**HR**	**SE**	***P*** ** value**	**HR**	**SE**	***P*** ** value**	**HR**	**SE**	***P*** ** value**	**HR**	**SE**	***P*** ** value**
Women age at marriage (yr)	0.69	0.01	0.001	1.01	0.01	0.213	1.02	0.02	0.318	0.96	0.03	0.282
Women's Education:												
Illiterate or primary	1.00			1.00			1.00			1.00		
Secondary	1.24	0.16	0.185	0.49	0.16	0.001	0.72	0.20	0.107	0.51	0.32	0.035
High school	1.14	0.51	0.277	0.42	0.20	0.001	0.34	0.32	0.001	0.67	1.03	0.996
Academic	0.97	0.23	0.905	0.42	0.24	0.001	0.19	0.51	0.001	0.43	1.17	0.469
Women's employment												
Employed	1.00			1.00			1.00			1.00		
Unemployed	0.68	0.03	0.005	1.05	0.15	0.725	1.23	0.39	0.586	1.67	0.88	0.557
Husband’s age												
Per one yr increase	1.01	0.01	0.203	0.99	0.01	0.117	0.98	0.01	0.109	1.01	0.02	0.711
Husband’s education												
Illiterate or primary	1.00			1.00			1.00			1.00		
Secondary	0.85	0.18	0.359	0.98	0.18	0.913	0.64	0.21	0.034	1.21	0.34	0.578
High school	0.70	0.21	0.088	0.87	0.21	0.516	0.63	0.33	0.165	1.03	0.81	0.974
Academic	0.74	0.22	0.183	0.93	0.22	0.755	0.87	0.34	0.680	1.05	0.88	0.955
Husband’s employment												
Employed	1.00			1.00			1.00			1.00		
Unemployed	0.88	0.04	0.039	0.59	0.33	0.111	1.37	0.40	0.423	0.66	1.03	0.682
Stillbirth												
Yes	1.00			1.00			1.00			1.00		
No	1.21	0.19	0.322	0.87	0.21	0.510	0.69	0.28	0.187	0.61	0.51	0.335
Maternal age at 1st pregnancy												
≤18 yr	1.00			1.00			1.00			1.00		
>18 yr	0.93	0.10	0.478	0.89	0.10	0.290	0.62	0.15	0.001	0.73	0.29	0.266
Family income (US$)												
≤135	1.00			1.00			1.00			1.00		
135-270	0.91	0.12	0.301	1.03	0.51	0.412	0.98	0.41	0.378	0.83	0.29	0.274
≥270	1.24	0.80	0.487	1.66	0.76	0.591	1.04	0.12	0.185	0.88	0.76	0.280

Women's education was the only variable that had a significant effect on the birth interval from the first to the second delivery, so that the hazard ratio at the second birth reduced with increasing mother education. The parent’s education had significant effects on the time interval between the second and the third deliveries. The hazard ratio of the third birth decreased sharply when maternal education increased. The hazard ratio of the third delivery in women, who had age at the first pregnancy above 18 yr decreased (Table 2). The women's education also had a significant effect on the birth interval from the third to the fourth delivery, such that the hazard ratio of the fourth delivery decreased in accordance with levels of education increased.

Table 3 shows the parity progression ratios (i.e., the chance of women with a certain number of children, e.g.,* i*^th^ children, who go onto have another child, e.g., *(i+1)*^th^ children) of childbirth among the women. The parity progression ratios were high since marriage to the first birth (0 to 1^st^ child), while these ratios has been fallen from the third birth and more.

**Table 3:** Estimates of parity progression ratios for different parities and according to six different values of C

**C value**	**Parity progression ratios (**αi**)**			
	**0 to 1** ^st^ ** child**	**1** ^st^ ** to 2** ^nd^ ** child**	**2** ^nd^ ** to 3** ^rd^ ** child**	**3** ^rd^ ** to 4** ^th^ ** child**
5 years	0.918	0.820	0.557	0.307
6 years	0.925	0.832	0.568	0.315
7 years	0.944	0.849	0.574	0.347
8 years	0.967	0.851	0.580	0.351
9 years	0.981	0.854	0.596	0.361
10 years	0.983	0.861	0.592	0.354

The study obtained estimates of the parity progression ratios (i.e., median of PPRs for different values of C) for parities one to four as 0.955, 0.838, 0.557, and 0.349, respectively.

The chance of the first to the fourth birth was decreasing, such that these chances for the third and the fourth children after 10 yr were 0.592 and 0.354, respectively (Table 3).

## Discussion

Fertility status has direct association on the structure and pyramid of population^6^. The number of parity depends on the fertility rate and the number of women in reproductive age^14, 15^. In addition, the fertility rate is dependent on cultural, social and economic of societies^14, 15^. Therefore, knowing of fertility status and birth spacing are important for policy makers at all fields (e.g., economic, population, health, etc.). In our study, women 45 yr or older were selected because they passed their reproductive age.

The sex ratio is a useful index to family programming, and was about 104 at the first birth in Hamadan. The sex ratio in 2015 (i.e., the present study) is similar to 2010 in Hamadan, but it is higher than in Iran (i.e., approximately 103)^8^. One of the most important results in this study was high spacing (e.g., ≥2 yr) between deliveries among Hamadan women. The evidence has indicated that a birth interval of more than 2 yr gives a better chance of infant survival^16^. In this study, not only the first birth spacing was more than 2 yr, but also the median time of the second, the third and the fourth deliveries was 4.53, 4.65 and 5.27 yr, respectively. Birth interval in Hamadan was larger than WHO recommendation^17^. Furthermore, developing countries have shorter birth intervals, while women in Hamadan (west of Iran) had large birth intervals^18^. Therefore, a decline in the population growth in Hamadan may be due to the large birth intervals.

In the study, we showed that maternal age at marriage had relation with the first birth spacing the same as previous studies^9, 19^, but unlike them we have shown that it had a negative relationship with the first birth spacing. Because the maternal age at marriage and their education level are concordance, hence, the negative relationship between maternal age at marriage and the first birth spacing may be due to women continue education after marriage.

In addition, we showed that employment status of women and their husbands had a negative relationship with the first birth spacing, in consistent with Begna et al^20^. Neither employment status of maternal nor employment status of their husbands had significant effect on the second and more birth spacing. In this study, we obtained parity progression ratios. It seems that chance of parity progression of the third to the fourth child and more are relatively low, and may be due to the implementation of family planning or increasing use of contraceptives in the past few decades^7, 19, 21^.

The relationship between stillbirth and birth interval was observed^22^, while we could not found it. Ethiopian women whose previous sibling had died had twelve months shorter birth interval^22^.

This research shows that parity progression ratio at 5-yr period from marriage to the first birth is 0.91, which is high. Such results show a delay in childbearing after marriage, despite the family wishes to have a first child. Delay in childbearing may be due to the socioeconomic situation couples, social support, continuing education of parents, or family support of couples. Of course, we are not sure. Therefore, we suggest that a study is done on the causes and factors associated with delayed childbearing.

As shown, parity progression ratios 1^st^ to 2^nd^ and 2^nd^ to 3^rd^ children at 5-yr period was 0.82 and 0.557, respectively and the subsequent birth had fallen. Hence, the average family size has reduced in the area. According to McDonald et al^23^, parity progression ratio from the third birth and more is highly reduced in Iran same the present study. Therefore, a national commitment is needed to prevent the continuation of this negative trend.

Detailed results of census 2010 showed that Hamadan had the lowest average annual population growth after Ardabil, Ilam, Lorestan and Gilan over the 2005-2010^8^. The results of this study and previous census shows that some population changes (e.g., growth of birth and family size) in Hamadan and Iran are the same during the last few decades^8, 23^, so that the average of family size in both Hamadan and Iran was reduced. However, it seems that Hamadan the same as Iran^23^ is experiencing transition and in both population growths is declining in the past few decades.

The study shows that chance of delivery at the third child and more have been declining in the past years in Hamadan. Accordingly, employment and education of parent may be affecting to the declining. This process will cause many problems such as aging population and increasing actual depending rate. In addition, the results showed that the chance of the second and the third birth, after at 10-yr period of marriage is reduced severity. With just a glance, it clearly observed that the decline in population growth occurred in Hamadan, west of Iran, and most families have been turn to one or two children.

Since the number of women having five or more children was small in this study, hence we limited the analysis up to fifth birth order.

## Conclusions

Reduced population growth and childbearing has been occurred since few decades ago in Hamadan. The main obstacle to reaching the third birth and more was the parents' education level in Hamadan.

## Acknowledgment

Hamadan University of Medical Sciences financially supported this research. The authors would like to thank the women and interviewers.

## Conflict of interest statement

No conflict of interest is declared.

## Funding

The study was funded by Vice-chancellor for Research and Technology, Hamadan University of Medical Sciences (No. 9104131928).

## Highlights

- Birth spacing in Hamadan is more than WHO recommendation
- Chance of childbearing in Hamadan have dramatically decline from the third child and then.
- One of the main causes of the redaction is maternal education level and employment of couple.
